# Rare Earth Element Characteristics of Shales from Wufeng–Longmaxi Formations in Deep-Buried Areas of the Northern Sichuan Basin, Southern China: Implications for Provenance, Depositional Conditions, and Paleoclimate

**DOI:** 10.1021/acsomega.3c03086

**Published:** 2024-01-01

**Authors:** Bin Xiao, Dongxu Guo, Sheng Li, Shuzhen Xiong, Zhaoyi Jing, Mingfei Feng, Xiang Fu, Zhonghai Zhao

**Affiliations:** † College of Mining, Liaoning Technical University, Fuxin 123000, China; ‡ Liaoning Geology Engineering Vocational College, Dandong 118000, China; § College of Environmental Science and Engineering, Liaoning Technical University, Fuxin 123000, China

## Abstract

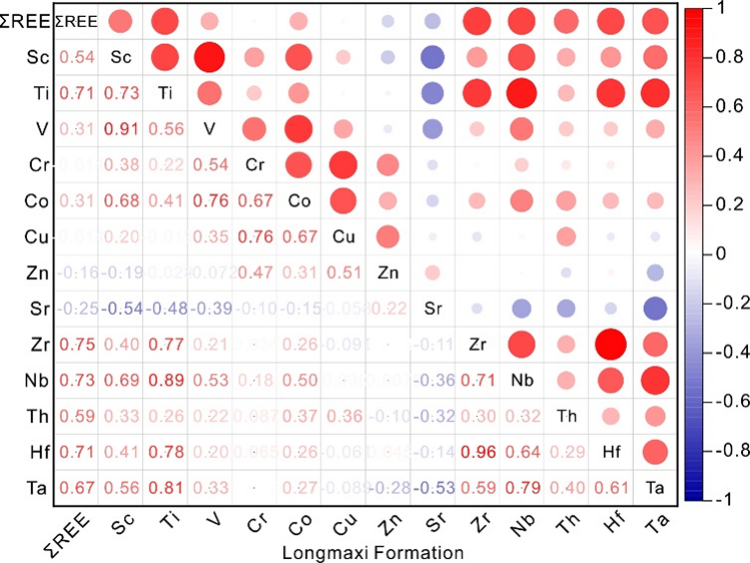

To explore the sedimentary environment and the background of the source area of organic-rich shales in the Wufeng–Longmaxi Formations in the northern Sichuan Basin, samples from Well XX1 in the area were subjected to geochemical testing and analysis of organic carbon content, trace elements, and rare earth elements (REEs). The results show that the total content of REE (ΣREE) of the shale in the Wufeng–Longmaxi Formations varied from 183.08 to 234.66 μg/g with an average of 212.59 μg/g, which is significantly higher than the content of the North American shale composite. The fluctuations in the total amount of REEs in the shale of the Wufeng–Longmaxi Formations reflect certain differences in the geochemical conditions of the Upper Ordovician–Lower Silurian shale. The ratios of LREE/HREE, La_N_/Yb_N_, La_N_/Sm_N_, and Gd_N_/Yb_N_ and the distribution of normalized REE patterns indicate that the source supply or sedimentary structural background may have changed during the shale deposition period of the Wufeng Formation, while the shale deposition period of the Longmaxi Formation may be in a relatively stable source supply and sedimentary structural background. There is no significant correlation between δCe and ΣREE, and the obviously negative Eu abnormity and the weak Ce abnormity indicated that the diagenesis had a limited impact on REEs. Geochemical parameters such as values of ∑REE, δEu, δCe, Ce_anom_, and La_N_/Yb_N_ indicate that the climate during the Wufeng–Longmaxi Formation shale deposition period was warm and humid, and the shale was deposited mainly in the suboxic-anoxic water environment. The deposition rate was stable and slow, providing good conditions for the production and preservation of organic matter. At the same time, this shows that the water environment of Wufeng Formation is more anoxic and reductive than that of Longmaxi Formation, which is more conducive to the preservation of organic matter. The correlation between ΣREE and the content of Sc, Ti, Cr, Co, Zr, Nb, Th, Hf, Ta, and other elements indicates that the sources of REEs in the shale of Wufeng and Longmaxi Formations in the study area are similar, mainly terrestrial clasts, and some may come from the sea. The REE distribution pattern shows that the shale provenance of the Wufeng–Longmaxi Formations mainly comes from the upper crust. The La/Yb–∑REE diagram shows that the sediment-parent rocks are mainly early sedimentary rocks and these sediment-parent rocks have granite provenance characteristics. Compared to La/Yb, LREE/HREE, La_N_/Yb_N_, and other REE characteristic parameters, it is inferred that the tectonic background of the study area is dominated by passive continental margin.

## 1 Introduction

The end of the Ordovician and the beginning of the Silurian was an important period in Earth history,^1−4^ and it is marked by marine mass extinction, large-scale glaciation, sea-level change, extensive volcanism, ocean anoxic event, and extensive deposition of marine black shale.^5−10^ Black marine shale, as a source rock for oil and gas, is widely accumulated in paleogeographic environments.^11−16^ To this day, such a black shale has been widely developed as unconventional reservoirs in many countries, such as the United States, Canada, Australia, China, Germany, Russia, etc.^17−22^ Thus, the study of the sedimentary environment and the mechanism of organic matter accumulation for the marine shales of the Upper Ordovician Wufeng Formation and the Lower Silurian Longmaxi Formation in the northern Sichuan Basin not only can provide important information about the climatic and paleoenvironmental change of the Ordovician–Silurian but also provides evidence for the prediction of favorable areas for oil and gas exploration.

In recent years, with increasing demand for the national production of natural gas, the intensity of unconventional gas exploration and development has been increasing. As one of the largest oil and gas basins in China, the Sichuan Basin is rich in natural gas resources, and remarkable achievements have been made in the exploration and development of unconventional gas in this basin.^22,23^ The shales of the Upper Ordovician Wufeng Formation–Lower Silurian Longmaxi Formation are the only shale formations in China from which shale gas has been commercially developed. From 2012 to 2022, shale gas fields such as Fuling, Weirong, Weiyuan, Changning, and Zhaotong fields have been discovered in these formations in the Sichuan Basin and surrounding areas.^23−25^ These formations have proven geological reserves of 10,455 × 10^8^ m^3^ and a shale gas productivity of 200 × 10^8^ m^3^ per year.^23−25^ Previous researchers have conducted extensive research on paleontology, paleogeography, paleoclimate, and paleomarine environment and sources of rock formation mechanisms of the Late Ordovician Early Silurian in the Sichuan Basin and have also gained many beneficial insights. This has laid a solid foundation for revealing the development mechanism of organic-rich shales.^3,4^ In addition, a large amount of research has been conducted on the main controlling factors for the enrichment of organic matter in shales, including changes in relative sea level, paleoproductivity, redox conditions, input of terrigenous debris, turbidity current, deposition rate, degree of water retention, volcanic activity, etc.^8−10^ Some scholars believe that the deposition of organic-rich shales is mainly controlled by the degree of retention of the basin, the high paleoproductivity, and the anoxic water. Some scholars also believe that the deposition of this set of shales is the result of the synergistic effect of paleoclimate and paleoceanic factors caused by geological time such as volcanic eruption, undercurrent intrusion, and terrigenous detrital input.^10,13,16^ In summary, there is still a great deal of controversy regarding the formation environment of the Wufeng–Longmaxi Formation shale, and there is also less research on the source background. Currently, previous studies on the geochemical characteristics of the Wufeng–Longmaxi Formation black shales buried less than 3500 m have been conducted in depth.^26−31^ However, due to the lack of deep drilling in the deep-buried areas of the Wufeng–Longmaxi Formations in the Sichuan Basin, representative samples cannot be collected systematically, which leads to the relatively slow evaluation of deep shale gas resources in deep-buried areas.

The drilling of some important wells has provided updated and more accurate geochemical data to study the shale of Wufeng–Longmaxi Formations. In order to understand the geological conditions of the occurrence of shale in Wufeng–Longmaxi Formations in the northern Sichuan Basin, based on the results of the rare earth and trace element testing in Well XX1, the geochemical element differences of shale in Wufeng–Longmaxi Formations were analyzed, and changes in sedimentary environment, source properties, and tectonic background were explored. This helps to understand the mechanism of shale formation in Wufeng–Longmaxi Formations and provides a certain geological basis for the subsequent exploration and development of shale gas in the northern region of the Sichuan Basin. At the same time, this also provides basic research data for the study of the global sedimentary-tectonic pattern during the Ordovician–Silurian transition period.

## 2 Geological Settings

**Figure 1:**
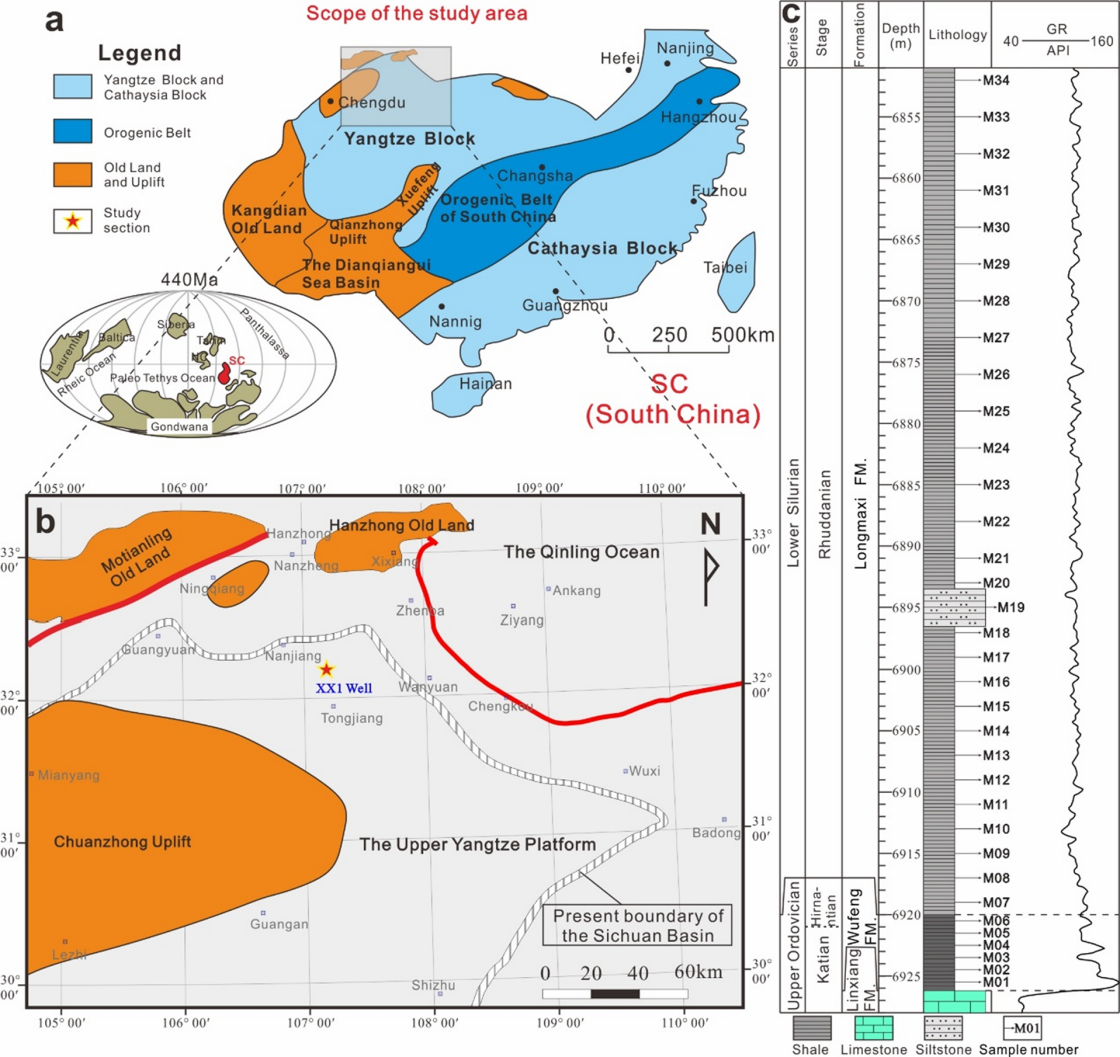
Paleogeographic location of the study area. (a) Relative position of the Yangtze Block in the Late Ordovician and its present position. The illustration in the lower left corner shows the relative position of South China on the global paleogeographic map reconstructed by predecessors. (b) Tectonic framework in the northern part of the Upper Yangtze Platform in the Late Ordovician and the location of the XX1 Well in Tongjiang, Sichuan Province. (c) Stratigraphic column of the Wufeng and Longmaxi Formations of the XX1 Well.

In the early Paleozoic Ordovician and Silurian transition period, the South China plate separated from the Gondwana continent but remained attached to the ancient equator in the northwest margin of the Gondwana continent (Figure 1a).^32,33^ The South China plate is composed of Yangtze and Cathaysia blocks (Figure 1a).^19,34^ From the Late Ordovician to the Early Silurian, the Caledonian movement was the most intense period. The Upper Yangtze Platform was in a compressive state, and many uplifts began to rise around the Platform, such as the “Chuanzhong uplift” in the northwest direction, the Yunnan-Guizhou old land in the south (“Qianzhong uplift”), and the southeast Jiangnan-Xuefeng uplift.^34^ Surrounded by these old land and uplifts, the Upper Yangtze Platform evolved in the Middle Ordovician sea area with extensive characteristics into the semilimited sea area adjacent to the northern part of the Qinling Sea in the Late Ordovician, forming a large area of low-energy, under-compensated, and anoxic sedimentary conditions (Figure 1b).^10,35^

The Sichuan Basin is located on the Upper Yangtze Platform.^36^ Well XX1, whose geological location is in Tongjiang County, Bazhong City, Sichuan Province, is located in the northern part of the Sichuan Basin (Figure 1b). This well is a pre-exploration well, with a drilling stratum of the Suining Formation (J_3_s) in the Upper Jurassic of the Mesozoic Era and a completion stratum of the second member of the Dengying Formation (Z_2_dy) in the Upper Sinian of the Proterozoic Era. The well was completed in 2016 with a completion depth of 8418 m, making it the deepest well in Asia at that time. The Upper Ordovician Wufeng Formation–Lower Silurian Longmaxi Formation is fully developed and buried at depths below 6500 m. The Wufeng Formation is a set of black shales. The Longmaxi Formation is integrally covered on the Wufeng Formation. The bottom of the Longmaxi Formation is composed of gray black and dark gray shales mixed with dark gray siltstone, and the upper part is composed of gray shales, silty mudstone mixed with light gray siltstone, and argillaceous siltstone (Figure 1c).

## 3 Materials and Methods

This study collected a total of 34 cutting samples from Wufeng–Longmaxi Formations in Well XX1. Among them, there are 6 black shale samples from the Wufeng Formation (samples M01-M06), 20 gray-black shale samples, 1 gray siltstone sample, and 7 gray shale samples from the Longmaxi Formation (samples M07 to M34). The sampling interval for cuttings is 1.0–3.0 m. The specific sampling location is shown in Figure 1c.

The trace and rare earth element analysis was completed at the Institute of Tibetan Plateau Research, Chinese Academy of Sciences. The acid solution method was adopted, and a Thermo Fisher X Series ICP-MS instrument was used. The detection limits for trace elements were 0.*n* × 10^–12^–*n* × 10^–12^ (*n* = 1–9). The analytical error of the element concentrations is generally better than ±0.5%.^37^ The specific treatment process was as follows. Approximately 10 g of sample was ground and dried in an oven at 55 °C for 12 h. After drying, 20.0 to 25.0 mg of sample was weighed in a Teflon container and wetted with a small amount of ultrapure water. Additionally, a blank sample and a standard reference sample (AGV-2) were prepared by using the entire process. Next, 1.0 mL of HNO_3_ and 1.0 mL of HF were added to the 10.0 mL Teflon container, and the mixture was ultrasonicated for 20 min. The Teflon container was then placed in a stainless-steel bomb. After tightening, the plate was placed in an oven and heated at 190 °C for 40 h. After being removed from the oven and cooled, the Teflon container was carefully removed from the stainless-steel bomb and heated on a heating plate at 150 °C until almost dry. Then, 1.0 mL of HNO_3_ was added, and the sample was heated until nearly dry. This process was repeated twice to ensure the removal of all of the HF. After completion, 2.0 mL of HNO_3_ and 3.0 mL of ultrapure water were added to the Teflon container, the Teflon container was placed in a stainless-steel bomb, and the bomb was tightly sealed. The bomb was then placed in an oven at 150 °C for more than 24 h. The dissolved sample was removed and the volume was adjusted to about 2000 times the weight of the sample. The element concentrations were determined by ICP-MS.^38^

The total organic carbon (TOC) content was analyzed at the Beijing Research Institute of Uranium Geology. The washed sample was ground to 80 mesh using an agate mortar, and then, 100 mg of the sample was weighed and dissolved in 5% of dilute hydrochloric acid multiple times until no bubbles were produced. The sample was soaked for 24 h to remove carbonate and other inorganic carbon. The test was then conducted with a German Eltra CS580A carbon and sulfur analyzer. According to repeated analysis of standard samples, the accuracy of the TOC content analysis is better than ±0.5%.^39^

## 4 Results

Based on the content of rare earth elements in the shale of the Upper Ordovician Wufeng Formation–Lower Silurian Longmaxi Formation of Well XX1 (Table 1), the parameters of rare earth elements, which reflect geochemical characteristics, can be calculated (Table 2). The parameters of rare earth elements can well reflect the characteristics of rare earth elements, different parameters characterizing the enrichment, and sources of different rare earth elements. The total amount of rare earth elements ∑REE in the shale of the Wufeng–Longmaxi Formations in the northern Sichuan region is 183.08–234.66 μg/g, with an average value of 212.59 μg/g, significantly higher than the average value of the North American shale composite (NASC) (173.21 μg/g),^40^ indicating that the shale of the Wufeng–Longmaxi Formations of Well XX1 in North Sichuan has a high content of rare earth elements. The ∑REE of the Wufeng Formation is 183.08–227.21 μg/g, with an average value of 199.64 μg/g. The ∑REE of the Longmaxi Formation is 184.81–234.66 μg/g, with an average value of 215.36 μg/g. The fluctuation of the total REE content in the Wufeng–Longmaxi Formation shale reflects that the geochemical conditions of the Upper Ordovician–Lower Silurian shale are different to some extent.

**Table 1:** Rare Earth Element (REE) Contents of the Wufeng–Longmaxi Formation Shale in Well XX1^a^

samples	La	Ce	Pr	Nd	Sm	Eu	Gd	Tb	Dy	Ho	Er	Tm	Yb	Lu	Y
M01	43.64	78.52	8.32	30.66	5.90	1.39	6.10	0.90	5.47	1.09	3.22	0.45	2.99	0.43	31.95
M02	42.14	77.44	8.09	29.72	5.66	1.50	5.50	0.79	4.83	0.96	2.88	0.41	2.76	0.40	27.95
M03	45.42	84.18	8.82	32.26	5.85	1.45	5.16	0.76	4.65	0.92	2.72	0.39	2.63	0.39	26.59
M04	52.27	98.23	10.41	37.82	6.79	1.60	5.77	0.89	5.22	1.04	3.16	0.46	3.11	0.45	30.12
M05	42.19	79.15	8.52	31.75	6.02	1.56	5.50	0.75	4.58	0.91	2.76	0.41	2.71	0.40	26.17
M06	47.97	92.28	10.27	38.66	7.04	1.57	5.53	0.77	4.55	0.90	2.70	0.39	2.67	0.39	25.47
M07	41.73	78.67	8.34	30.77	5.89	1.55	5.14	0.77	4.62	0.93	2.83	0.41	2.76	0.40	26.29
M08	46.97	87.84	9.03	33.27	6.31	1.75	5.63	0.81	4.87	0.99	2.93	0.43	2.81	0.42	27.54
M09	50.34	95.83	10.06	37.69	7.15	1.73	6.06	0.88	5.18	1.03	3.10	0.44	2.99	0.45	28.08
M10	48.68	89.19	9.18	33.51	6.30	1.67	5.49	0.81	4.95	1.00	3.01	0.44	2.95	0.44	27.76
M11	48.32	94.13	9.96	36.79	6.94	1.65	5.97	0.91	5.39	1.08	3.21	0.46	3.10	0.45	29.63
M12	47.90	93.45	9.85	36.38	6.96	1.55	6.07	0.92	5.62	1.12	3.32	0.48	3.15	0.47	30.93
M13	50.92	101.50	10.77	39.90	7.43	1.68	6.11	0.91	5.31	1.06	3.18	0.46	3.06	0.45	29.47
M14	47.54	90.88	9.48	35.02	6.64	1.59	5.84	0.88	5.33	1.08	3.23	0.46	3.11	0.46	29.77
M15	47.33	91.66	9.55	35.18	6.66	1.62	5.79	0.87	5.27	1.06	3.18	0.46	3.12	0.46	29.62
M16	47.01	90.62	9.79	36.10	6.82	1.62	5.71	0.87	5.25	1.06	3.16	0.46	3.12	0.45	28.83
M17	49.89	94.89	9.89	36.43	6.91	1.55	6.00	0.89	5.42	1.09	3.22	0.47	3.15	0.46	29.97
M18	53.50	97.55	10.07	36.60	6.81	1.61	5.74	0.86	5.24	1.04	3.12	0.46	3.09	0.45	29.20
M19	47.69	92.13	9.60	35.46	6.75	1.52	5.80	0.91	5.33	1.07	3.18	0.46	3.10	0.45	29.37
M20	48.56	92.77	9.61	35.37	6.75	1.66	5.84	0.89	5.30	1.07	3.18	0.46	3.12	0.45	29.94
M21	48.36	93.82	9.96	37.08	7.10	1.68	6.12	0.93	5.48	1.10	3.28	0.47	3.11	0.47	30.17
M22	45.98	88.29	9.41	34.99	6.65	1.62	5.78	0.86	5.17	1.03	3.14	0.44	3.05	0.45	28.83
M23	50.53	94.96	9.88	36.73	6.88	1.68	5.86	0.91	5.38	1.08	3.21	0.47	3.17	0.47	29.97
M24	48.26	88.81	9.39	35.34	6.66	1.86	6.08	0.87	5.28	1.05	3.18	0.45	3.07	0.45	29.51
M25	46.39	89.94	9.38	34.93	6.79	1.74	6.20	0.92	5.50	1.10	3.25	0.47	3.14	0.46	30.84
M26	47.75	91.56	9.62	35.75	6.91	1.69	6.07	0.91	5.48	1.11	3.26	0.46	3.10	0.46	30.48
M27	50.45	96.30	9.96	36.95	7.16	1.83	6.15	0.91	5.43	1.09	3.30	0.46	3.19	0.47	30.60
M28	47.77	92.15	9.87	36.30	6.89	1.76	6.04	0.90	5.43	1.08	3.21	0.47	3.17	0.46	29.85
M29	54.90	100.70	10.52	38.40	7.22	1.82	6.22	0.91	5.47	1.10	3.28	0.47	3.20	0.47	30.78
M30	47.66	90.74	9.53	35.34	6.85	1.67	6.11	0.88	5.43	1.10	3.25	0.47	3.23	0.47	30.43
M31	48.58	92.76	9.64	35.54	6.80	1.63	5.90	0.90	5.36	1.08	3.13	0.46	3.08	0.45	29.67
M32	47.29	91.34	9.71	36.13	6.96	1.65	5.98	0.90	5.47	1.08	3.23	0.47	3.15	0.46	30.24
M33	49.37	93.89	9.98	37.03	7.01	1.66	6.10	0.91	5.53	1.11	3.33	0.48	3.24	0.47	30.72
M34	47.76	91.02	9.76	36.29	6.94	1.58	5.94	0.90	5.37	1.08	3.25	0.47	3.16	0.46	29.86
NASC^40^	32	73	7.9	33	5.7	1.24	5.2	0.85	5.8	1.04	3.4	0.5	3.1	0.48	27
chondrites^41^	0.367	0.957	0.137	0.711	0.231	0.087	0.306	0.058	0.381	0.0851	0.249	0.0356	0.248	0.0381	2.1

a The units of sample test results in the table are μg/g; samples M01-M06 are from the Wufeng Formation, and samples M07-M34 are from the Longmaxi Formation. NASC data are cited from Haskin et al.^40^ The rare earth element data of chondrites are cited from Taylor and Mclennan.^41^

**Table 2:** REE Geochemical Characters and TOC Contents of the Wufeng–Longmaxi Formation Shale in Well XX1^a^

samples	∑REE	L/H	La_N_/Yb_N_	La_N_/Sm_N_	Gd_N_/Yb_N_	δEu	δCe	La_n_/Yb_n_	Ce_anom_	TOC (%)
M01	189.07	8.16	9.86	4.66	1.65	0.71	0.97	1.41	–0.05	3.15
M02	183.08	8.88	10.32	4.69	1.62	0.82	0.98	1.48	–0.05	3.80
M03	195.61	10.10	11.67	4.89	1.59	0.81	0.99	1.67	–0.04	4.06
M04	227.21	10.31	11.35	4.85	1.50	0.78	0.99	1.63	–0.04	3.94
M05	187.20	9.39	10.53	4.41	1.65	0.83	0.98	1.51	–0.04	4.22
M06	215.66	11.06	12.16	4.29	1.68	0.77	0.97	1.74	–0.04	2.59
M07	184.81	9.35	10.23	4.46	1.51	0.86	0.99	1.47	–0.04	2.07
M08	204.06	9.80	11.29	4.69	1.62	0.90	1.00	1.62	–0.04	1.15
M09	222.92	10.08	11.38	4.43	1.64	0.80	1.00	1.63	–0.04	1.09
M10	207.61	9.88	11.15	4.86	1.51	0.87	0.99	1.60	–0.04	1.30
M11	218.37	9.61	10.54	4.38	1.56	0.78	1.01	1.51	–0.03	0.79
M12	217.25	9.27	10.28	4.33	1.56	0.73	1.01	1.47	–0.03	0.94
M13	232.74	10.33	11.24	4.31	1.62	0.76	1.02	1.61	–0.02	1.07
M14	211.51	9.38	10.33	4.51	1.52	0.78	1.00	1.48	–0.03	1.04
M15	212.20	9.50	10.24	4.47	1.50	0.80	1.01	1.47	–0.03	1.18
M16	212.05	9.56	10.19	4.34	1.48	0.79	0.99	1.46	–0.03	1.06
M17	220.26	9.64	10.69	4.54	1.54	0.73	1.00	1.53	–0.03	0.77
M18	226.14	10.31	11.70	4.94	1.51	0.79	0.98	1.68	–0.05	0.82
M19	213.44	9.51	10.41	4.45	1.52	0.74	1.01	1.49	–0.03	0.62
M20	215.03	9.59	10.53	4.53	1.52	0.81	1.01	1.51	–0.03	0.67
M21	218.96	9.45	10.52	4.29	1.60	0.78	1.00	1.51	–0.03	0.80
M22	206.87	9.39	10.18	4.35	1.54	0.80	0.99	1.46	–0.04	0.82
M23	221.20	9.76	10.76	4.62	1.50	0.81	1.00	1.54	–0.04	0.77
M24	210.75	9.31	10.64	4.56	1.61	0.89	0.98	1.52	–0.05	0.80
M25	210.20	9.00	9.99	4.30	1.60	0.82	1.01	1.43	–0.03	1.37
M26	214.12	9.27	10.40	4.35	1.58	0.80	1.00	1.49	–0.03	1.35
M27	223.65	9.65	10.68	4.43	1.56	0.84	1.01	1.53	–0.03	1.94
M28	215.51	9.38	10.18	4.36	1.54	0.83	0.99	1.46	–0.03	1.50
M29	234.66	10.12	11.61	4.79	1.58	0.83	0.98	1.66	–0.05	0.78
M30	212.70	9.17	9.97	4.38	1.53	0.79	1.00	1.43	–0.04	1.32
M31	215.32	9.57	10.65	4.50	1.55	0.79	1.00	1.53	–0.03	0.51
M32	213.81	9.31	10.16	4.28	1.54	0.78	1.00	1.46	–0.03	0.45
M33	220.10	9.40	10.28	4.44	1.52	0.77	0.99	1.47	–0.04	0.99
M34	213.97	9.37	10.22	4.33	1.52	0.75	0.99	1.47	–0.04	0.47

a The total rare earth elements ∑REE = LREE+HREE. The ratio of the total light rare earth element content to the total heavy rare earth element content L/H = LREE/HREE. The total light rare earth element contents LREE = La+Ce+Pr+Nd+Sm+Eu. The total heavy rare earth element contents HREE = Gd+Tb+Dy+Ho+Er+Tm+Yb+Lu; La_N_/Yb_N_, La_N_/Sm_N_, and Gd_N_/Yb_N_ are the ratios for the chondrite-normalized values; δEu = Eu_N_/(Sm_N_ × Gd_N_)^0.5^; δCe = Ce_N_/(La_N_ × Pr_N_)^0.5^. La_n_/Yb_n_ is the ratio for the NASC-normalized values; cerium anomaly index Ce_anom_ = lg[3(Ce_n_/(2La_n_+Nd_n_))].

**Figure 2:**
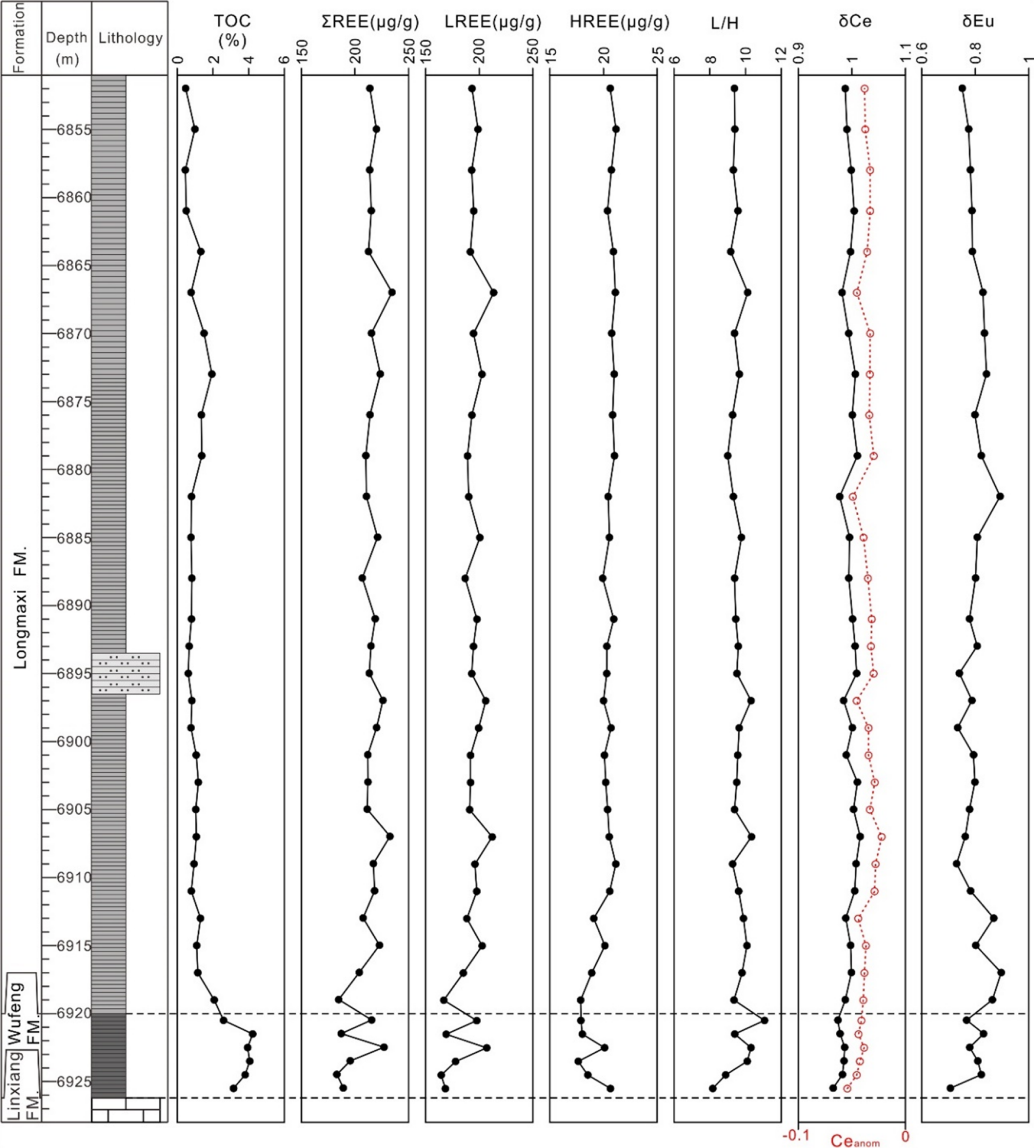
Vertical distribution of shale geochemical parameters in XX1 Well.

The ratio of the light rare earth element (LREE) content to the heavy rare earth element (HREE) content can effectively reflect the degree of differentiation between the light and heavy rare earth elements in the sample. The LREE contents in the study area range from 164.55 to 213.55 μg/g, with an average value of 192.49 μg/g, which is higher than the 152.84 μg/g of NASC-LREE. The HREE contents range from 17.63 to 21.17 μg/g, with an average value of 20.10 μg/g, which is basically consistent with the NASC-HREE of 20.37 μg/g. The ratio of LREE/HREE ranges from 8.16 to 11.06, with an average value of 9.58, which is much higher than the LREE/HREE value of 7.50 in the NASC,^40^ indicating a relative enrichment of light rare earth elements and a relative loss of heavy rare earth elements. The trend of vertical variation of the ∑REE and LREE contents in the shale of the Wufeng Formation is basically consistent from bottom to top, showing two cycles from low to high, while the HREE contents show two cycles from high to low. The overall trend of variation of the content of ∑REE, LREE, and HREE in the shale of the Longmaxi Formation is basically consistent from bottom to top. The lower part of the Longmaxi Formation presents two cycles from low to high, while the upper part of the Longmaxi Formation tends to be constant. This indicates that the provenance supply or sedimentary-tectonic setting may have changed during the sedimentary period of the Wufeng Formation shale, resulting in the opposite trend of variation of the LREE and HREE contents and the failure to maintain the same ratio.^10^ During the sedimentary period of the Longmaxi Formation shale, it may have been under a relatively stable provenance supply and sedimentary-tectonic background. Although the contents of ∑REE, LREE, and HREE vary from bottom to top, the ratio of LREE to HREE is relatively stable, maintaining the overall characteristics of light rare earth enrichment and heavy rare earth depletion (Figure 2).

The values of La_N_/Yb_N_, La_N_/Sm_N_, and Gd_N_/Yb_N_ are the slopes of the distribution curves of the chondrite-normalized REE patterns. It can be seen from the curve of chondrite-normalized REE patterns that the curve can be roughly divided into two sections, namely, the light rare earth element section and the heavy rare earth element section. Previous studies have suggested that the slope of the segment curve of light rare earth elements can be reflected by the La_N_/Sm_N_ ratio.^42,43^ The higher the value, the more enriched the light rare earth elements are and the higher the degree of fractionation between the light rare earth elements. Similarly, the slope of the curve for the heavy rare earth element segment can be reflected by the Gd_N_/Yb_N_ ratio, with a smaller value indicating a greater enrichment of heavy rare earth elements and a smaller degree of fractionation between heavy rare earth elements. The overall slope of the curve can be reflected by the La_N_/Yb_N_ ratio. If this value is greater than 1, the curve is tilted to the right, indicating enrichment of light rare earth elements.^42,43^ The La_N_/Yb_N_ values of samples from Well XX1 in the study area range from 9.86 to 12.16, with an average value of 10.66. The La_N_/Sm_N_ values range from 4.28 to 4.94, with an average of 4.50. The Gd_N_/Yb_N_ values range from 1.48 to 1.68, with an average value of 1.56. The results show that the light rare earth elements differ significantly between the Wufeng–Longmaxi Formation shale, while the differentiation of heavy rare earth elements between the Wufeng–Longmaxi Formation shale is not significant.^44,45^

δEu and δCe values are one of the important indexes of rare earth elements, which reflect the abnormal degree of Eu and Ce, respectively. Eu is a variable valence element with two valence states, Eu^2+^ and Eu^3+^. Under the condition of an oxidation environment, it remains in a positive trivalent state like other rare earth elements. If the catalyst is in the reducing environment, Eu^3+^ will be restored to Eu^2+^. Generally speaking, a δEu value greater than 1.05 is a positive anomaly, while a δEu value less than 0.95 is a negative anomaly.^45,46^ The δEu values of the samples in the study area range from 0.71 to 0.90, with an average value of 0.80, which is slightly higher than the δEu value of 0.70 in the NASC, showing a negative anomaly. Ce is affected by environmental redox conditions and pH changes, which cause it to have usually two valence states of Ce^3+^ and Ce^4+^. In the oxidizing environment, Ce^3+^ will be oxidized to Ce^4+^, resulting in an increase in the concentration of Ce^4+^ in sediments and an enrichment of Ce in sediments. Therefore, the Ce anomaly plays an indicative role in the analysis of the sedimentary environment and water medium conditions. The δCe values of the samples in the study area range from 0.97 to 1.02, with an average value of 0.99, which is slightly lower than the δCe value of 1.08 in the NASC, showing a weak negative anomaly to a weak positive anomaly and overall showing a weak negative anomaly. The vertical δEu and δCe values fluctuate in a small range, indicating that the water environment of the Wufeng–Longmaxi Formations was relatively stable during the sedimentary period.

**Figure 3:**
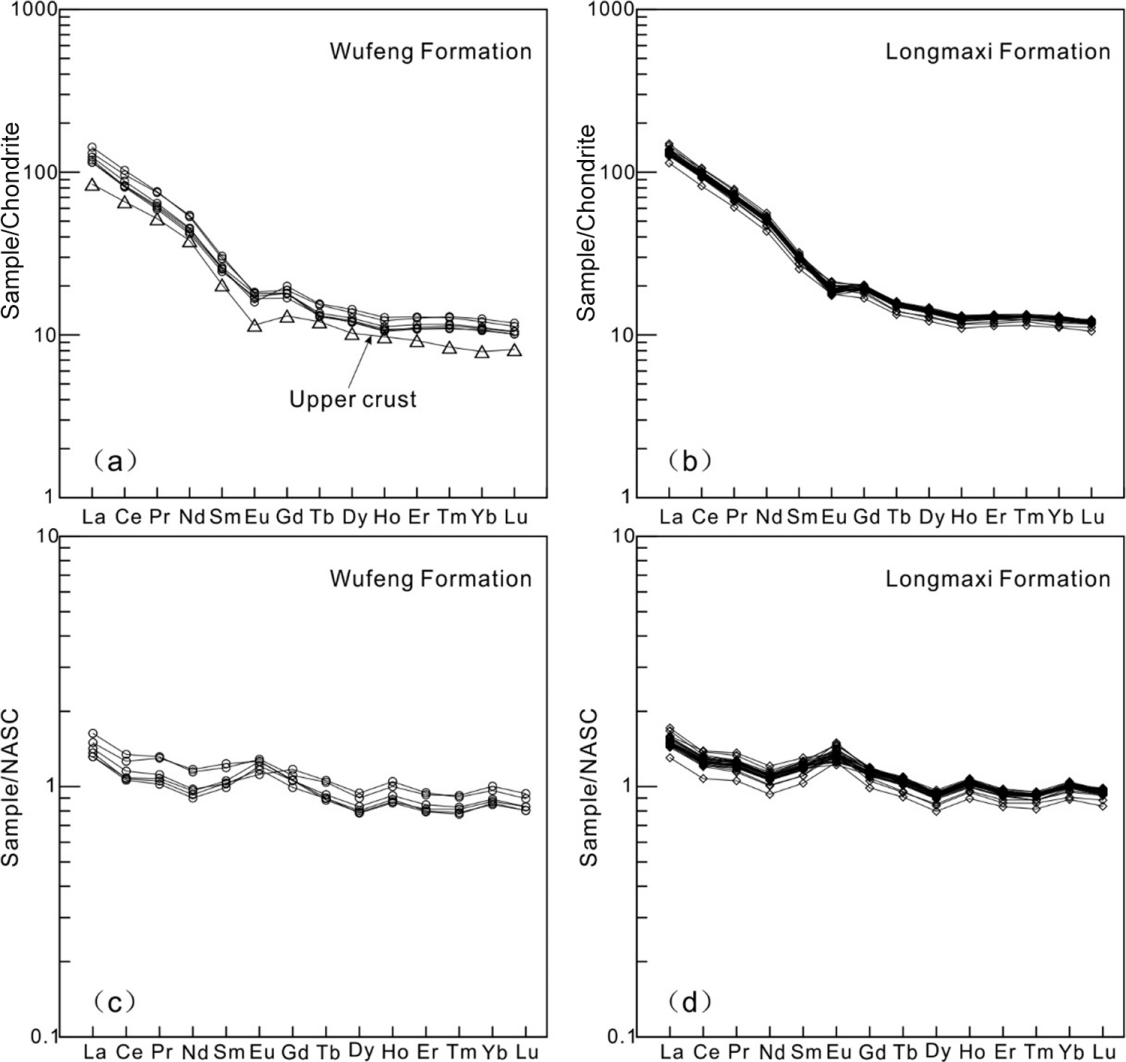
REE distribution patterns of shales in Well XX1. (a) Chondrite-normalized REE patters of the Wufeng Formation shale. (b) Chondrite-normalized REE patterns of the Longmaxi Formation shale. (c) NASC-normalized REE patterns of the Wufeng Formation shale. (d) NASC-normalized REE patters of the Longmaxi Formation shale.

At present, the distribution patterns of rare earth elements in shales mainly include two models: chondrite-normalized patterns and NASC-normalized patterns.^40,41^ Based on the test data, REE distribution patterns of shales in the Wufeng–Longmaxi Formations of Well XX1, including the chondrite-normalized and NASC-normalized, were drawn (Figure 3). The normalized pattern diagram of rare earth elements in chondrite shows that the distribution curves of rare earth elements in the shale of the Wufeng–Longmaxi Formations are moderately tilted to the right, indicating a significant differentiation between light and heavy rare earth elements. The content of light rare earth elements is enriched, and the content of heavy rare earth elements is stable, with significant negative Eu anomalies (Figure 3a,b). This distribution pattern is consistent with the distribution characteristics of REEs in the upper crust (Figure 3a,b),^47^ indicating that the shale source of the Wufeng–Longmaxi Formations in the northern Sichuan Basin mainly comes from the upper crust. The normalized pattern diagram of rare earth elements in the NASC shows that the distribution curves of rare earth elements in the Wufeng–Longmaxi Formation shale are generally nearly flat, showing a slight right-leaning feature, with obvious Eu positive anomalies (Figure 3c,d), indicating that the Wufeng–Longmaxi Formation shale is more enriched in Eu compared to the NASC, suggesting that its sedimentary water is more reduced or its provenance area is richer in Eu.

## 5 Discussion

### 5.1 Diagenetic Influence

**Figure 4:**
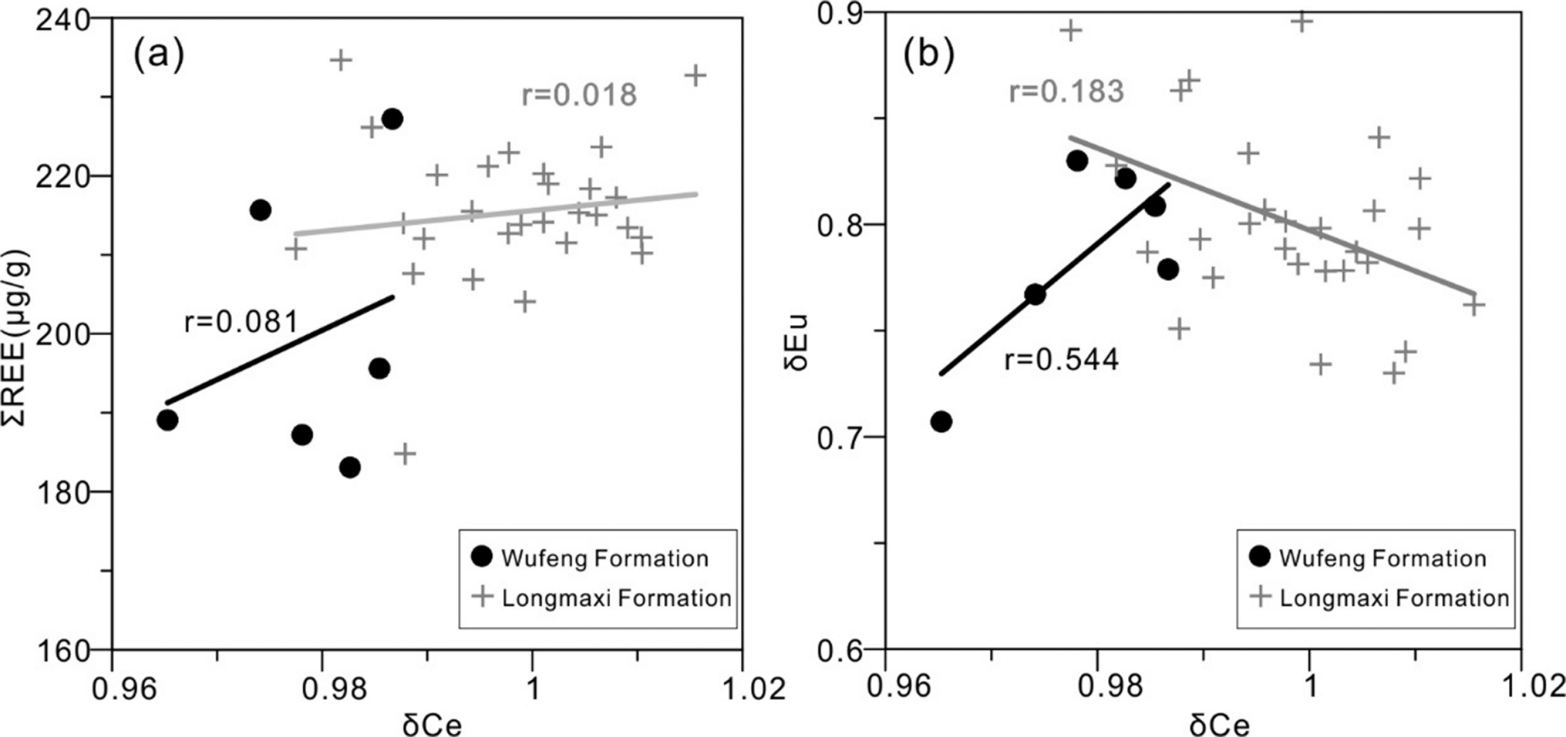
Diagrams of δCe vs ∑REE (a) and δCe vs δEu (b) for the Wufeng–Longmaxi Formation shale in Well XX1.

Before discussing the geological significance of the REE characteristics, it is necessary to first analyze the influence of diagenesis on the REEs in the samples. The vitrinite reflectance Ro of the Wufeng Formation shale of Well XX1 is 2.8%, while the vitrinite reflectance Ro of the Longmaxi Formation shale is 2.2%.^10^ Although there are few measuring points for vitrinite in the shale, combined with their current burial depth exceeding 6000 m, this indicates that the evolution of organic matter in the Wufeng–Longmaxi Formation shale has entered a stage of high maturity to overmaturity. Diagenesis has entered the phase of quasi-metamorphism.^48^ Therefore, it is necessary to discuss whether diagenesis has an impact on the REE characteristics. Shields and Stille proposed that diagenesis can change the abnormal Ce values, resulting in a good positive correlation between δCe and ∑REE and a good negative correlation between δCe and δEu.^49^ As shown in Figure 4a, there is no obvious correlation between δCe and ∑REE in the shale of the Wufeng–Longmaxi Formations. As shown in Figure 4b, δCe and δEu of the Wufeng Formation shale are positively correlated, with correlation *r* = 0.544. δCe and δEu of the Longmaxi Formation shale show a weak negative correlation, correlation *r* = 0.183, reflecting that diagenesis has a very limited influence on REE of the Wufeng–Longmaxi Formation shale.^44^ Therefore, the REE geochemical characteristics of the shale samples in the study area can basically reflect the original sedimentary geological characteristics.

### 5.2 Sedimentary Environment

The distribution characteristics of REEs in sedimentary rocks will be affected by the sedimentary environment; therefore, some REE parameters can be used to identify the sedimentary environment. It is believed that the ∑REE in sedimentary rocks can be used to indicate climate change, and it is generally believed that a higher total amount of REE indicates a warm and humid climate and vice versa.^44^ The ∑REE of the Wufeng–Longmaxi Formation shale is significantly higher than the average of the NASC (173.21 μg/g),^40^ indicating that the Wufeng–Longmaxi Formation shale had a warm and humid climate during deposition. Additionally, previous studies on sediments such as loess, lakes, and paleosoils have found that significant negative Eu anomalies generally indicate a warm and humid climate.^50,51^ The δEu values of the Wufeng–Longmaxi Formation shales in Well XX1 are generally less than 0.9 with an obvious negative Eu anomaly (Table 2), indicating that they were formed in a warm and humid climate, which is consistent with the conclusion of a higher total amount of REEs.

Furthermore, Ce, as a variable valence element, can reflect the redox conditions of sedimentary water bodies.^45^ Previous studies have shown that the δCe value is affected by the depth of water. The higher the δCe value is, the shallower the water and the richer the oxygen. On the contrary, the smaller the δCe value is, the deeper and more anoxic the water body will be.^52^ When the NASC is used as the standard, the cerium anomaly index (Ce_anom_) can also be used to judge the redox conditions of ancient water bodies. It is generally believed that Ce_anom_ > −0.1, indicating that the water bodies are anoxic reduction conditions, Ce_anom_ < −0.1, reflecting oxygen-rich oxidation conditions in water.^53^Table 2 shows that the δCe values of the shale samples from Wufeng–Longmaxi Formations in Well XX1 range from 0.97 to 1.01, with an average value of 0.99, showing a slight negative Ce anomaly, indicating that the water body was suboxic during deposition. The Ce_anom_ value of the samples ranges from −0.02 to −0.05, with an average value of −0.04, slightly higher than −0.1, which indicates that the sedimentary water of Well XX1 is under anoxic reduction conditions. It can be found from the vertical evolution diagram of the geochemical parameters of Well XX1 (Figure 2) that the δCe values of the Wufeng Formation range from 0.97 to 0.99, with an average value of 0.98, and the δCe values of the Longmaxi Formation range from 0.98 to 1.02, with an average value of 1.0. This indicates that the water environment during the deposition of the Wufeng Formation was more stable than that of the Longmaxi Formation. During the deposition of the Longmaxi Formation, the redox conditions of the water body experienced multiple fluctuations, which may have been affected by sea-level changes. At the same time, this shows that the water environment during the deposition of the Wufeng Formation was more oxygen-poor, with stronger reducibility and more conducive to the preservation of organic matter. This can also be seen in the TOC content, that is, the content of residual organic carbon (Table 2). The TOC content of the Wufeng Formation is 2.59–4.22%, with an average of 3.63%. The TOC content of the Longmaxi Formation is 0.45–2.07%, with an average value of 1.02%.

Trace elements V, U, and Mo all have variable valence states and are redox-sensitive elements. Their enrichment in sediments is controlled by environmental redox conditions.^54^ V (vanadium) concentrates preferentially in anoxic sediments.^55^ V migrates from water column to sediments in both nonsulfide anoxic and sulfide euxinic conditions. Under nonsulfide anoxic conditions, the reduction of V (V) to V (IV) is accelerated by humic acid and fulvic acid produced during the degradation of organic matter, so the abundance of V is usually correlate well with the TOC content.^55^ On the contrary, the reduction of V (IV) to V (III) does not depend on the organic reactions under sulfide euxinic conditions.^55^ U (uranium) exists mainly in the form of solubility of uranyl carbonate complexes [UO_2_(CO_3_)4-3] in oxic-to-suboxic seawater, but under certain reducing conditions, it can be reduced to U(IV) and precipitated into sediments in the form of crystalline uraninite (UO_2_) or its metastable precursors.^56^ Because bacterial sulfate reduction can reduce U(VI) to U(IV) to a certain extent, and the abundance of organic matter controls the strength of sulfate reduction activity, the enrichment of U is usually highly correlated with the TOC content under nonsulfide anoxic conditions.^54^ However, in the sulfide euxinic phase, the increase of U concentration is weakly correlated with the TOC content.^55^ Therefore, the content of these elements and the ratio of element pairs can serve as an important indicator for determining sedimentary environments.^54,55^ In order to eliminate the dilution effect of terrestrial debris flux on it, V/Al, U/Al, and Mo/Al were used to evaluate the paleoredox conditions of the study profile, as Al is typically derived from the primitive debris material.^55^ When the paleoredox conditions of the study profile were evaluated using V/Al, U/Al, and Mo/Al, it was found that the relative changes in vertical redox conditions can be identified on one profile, but the degree of their redox conditions cannot be qualitatively identified. Therefore, several commonly used element ratio discrimination indicators such as U/Th, V/Cr, and V/Sc were introduced to comprehensively evaluate the paleoredox conditions of the studied profile.^54,55^ Among trace elements, U has a variable valence state, while Th is not affected by the redox conditions. Therefore, the ratio of U to Th can be used as an indicator to distinguish between redox conditions. In general, the U/Th value less than 0.75 indicates oxidation conditions; U/Th values ranging from 0.75 to 1.25 indicate suboxic conditions; and the U/Th value greater than 1.25 indicates anoxic conditions.^56^ The enrichment degree of V can be corrected by the abundance of the Sc element. The V/Sc value is low in oxidizing environments, generally less than 9.1, but high in anoxic environments.^57^ Under anoxic conditions, V is more easily enriched than Cr in organic sedimentary rocks. Based on this, it is proposed that V/Cr can be used as a discriminant indicator for redox conditions. When V/Cr is less than 2, it indicates oxidation conditions, when V/Cr is 2–4.5, it indicates suboxic conditions, and when V/Cr is greater than 4.5, it indicates anoxic conditions.^56^

**Figure 5:**
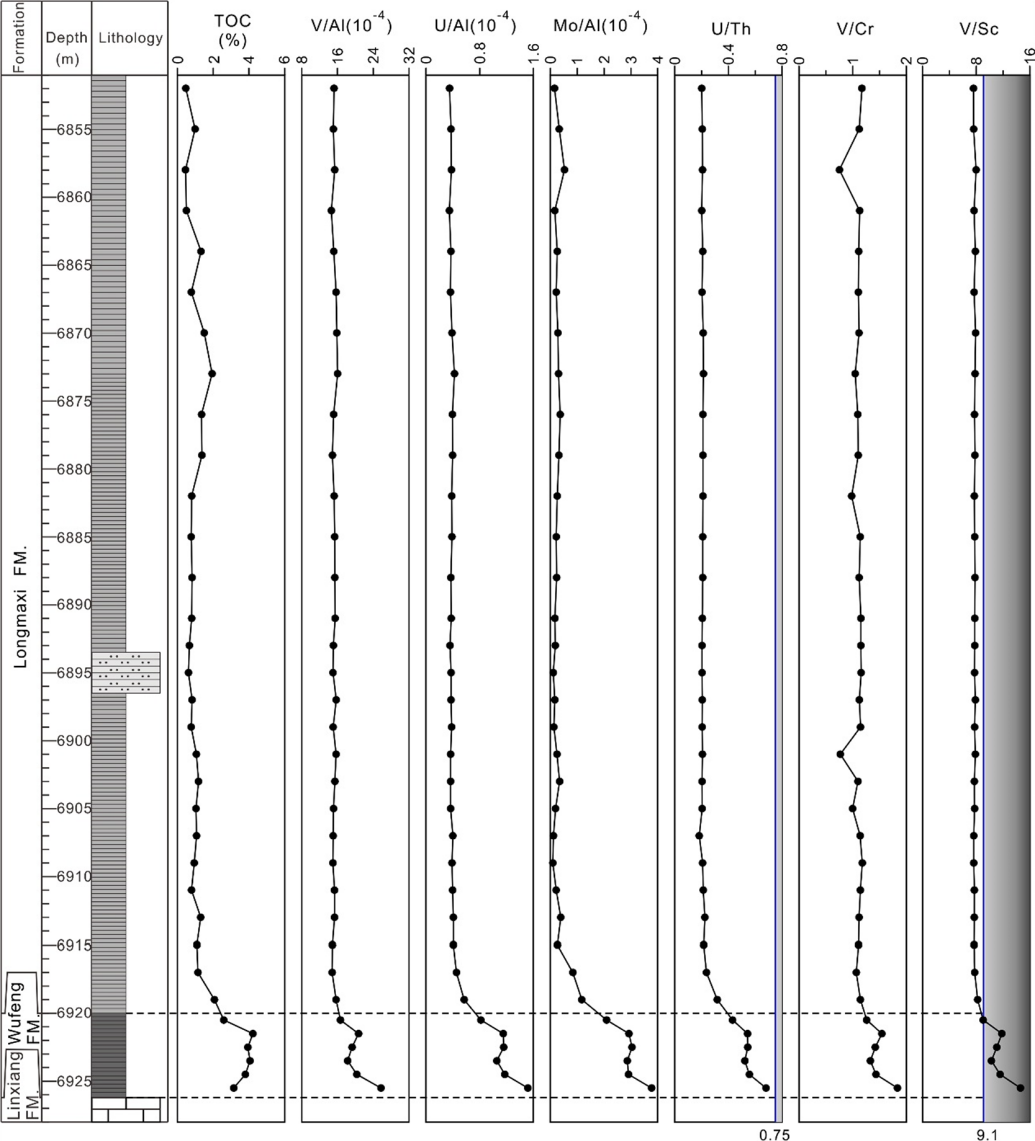
Vertical distribution of discriminant indicators for shale redox conditions in XX1 Well.

The Wufeng Formation shale from Well XX1 exhibits a downward decreasing trend in V/Al, U/Al, and Mo/Al. The V/Al, U/Al, and Mo/Al of the Longmaxi Formation shale show little variation (Figure 5). The similar trend of changes in the shale of the Longmaxi Formation depends on the migration and enrichment patterns of redox-sensitive elements in seawater, and on the other hand, it is related to the relatively stable redox conditions of seawater during its sedimentation period. The TOC content and redox condition indicators of the shale in the Wufeng–Longmaxi Formations of Well XX1 exhibit similar changing trends (Figure 5). The U/Th and V/Cr ratios of the Wufeng Formation shale exhibit oxidation conditions, while the V/Sc ratio exhibits suboxic conditions (Figure 5). Based on its gray black rock characteristics, it is believed that the suboxic-anoxic conditions are more consistent with the actual situation. The U/Th, V/Cr, and V/Sc ratios of the Longmaxi Formation generally exhibit oxidation conditions (Figure 5), but combined with their Ce_anom_ values and slightly lighter color characteristics compared to the shale of the Wufeng Formation, it is believed that the suboxic conditions are more consistent with the actual situation. The TOC content of the Wufeng–Longmaxi Formations is mainly controlled by the bottom water redox conditions.

Based on the above analysis, it is believed that the Wufeng–Longmaxi Formations in the XX1 Well area of the northern Sichuan Basin were in a suboxic-anoxic sedimentary environment, with a warm and humid ancient climate, providing good conditions for the production and preservation of organic matter.

### 5.3 Source of REEs

The source of REEs is studied by analyzing the correlation between the ∑REE and other elements. The correlation analysis between the ∑REE of the Wufeng–Longmaxi Formation shale and their trace elements with statistical significance and the results are shown in Table 3. Generally, among the trace elements that have statistical significance with ∑REE, it is generally believed that Sc, Zr, Nb, and Hf in sedimentary rocks come mainly from terrigenous debris,^58^ and the variation in the mass fraction of Ti, Cu, Zn, Nb, and Cr is mainly subject to the “element granularity control law”, mainly occurring in fine terrigenous debris.^46^

**Table 3:** Contents of Partial Trace Elements of Shales in XX1 Well and Correlation Coefficient with ∑REE^a^

samples	Sc	Ti	V	Cr	Co	Cu	Zn	Sr	Zr	Nb	Th	Hf	Ta
M01	10.77	2738.00	157.20	85.76	16.10	61.32	145.70	324.50	159.50	19.00	13.63	4.14	1.24
M02	11.98	3006.00	138.40	96.52	15.61	52.46	158.10	238.90	171.90	20.19	14.36	4.50	1.34
M03	12.42	3091.00	127.50	95.94	16.12	50.97	136.30	200.90	169.70	19.68	14.08	4.33	1.24
M04	12.24	3072.00	135.40	95.34	15.82	51.86	155.10	227.40	239.40	30.77	14.94	5.88	1.90
M05	12.18	3066.00	143.90	93.21	15.75	57.35	159.60	251.60	164.60	17.42	14.72	4.28	1.18
M06	14.31	3479.00	129.30	102.60	17.24	53.36	133.40	217.30	159.50	14.86	14.72	4.15	1.03
M07	15.72	3782.00	129.10	112.90	18.38	46.07	135.20	209.70	161.10	14.14	14.76	4.27	0.97
M08	14.88	4047.00	115.70	108.40	16.73	40.10	127.40	241.10	184.80	14.74	14.96	4.81	1.03
M09	16.18	4254.00	124.40	112.20	18.29	46.05	139.40	199.20	182.30	15.54	15.82	4.80	1.07
M10	16.76	4387.00	129.30	115.60	18.67	40.12	129.70	205.10	180.90	15.99	15.26	4.73	1.08
M11	16.97	4405.00	131.10	114.70	18.84	43.42	119.20	175.10	179.30	15.70	15.91	4.74	1.10
M12	17.16	4490.00	131.00	111.10	18.88	43.51	101.90	109.50	188.00	15.83	16.30	4.91	1.11
M13	17.06	4443.00	130.30	114.40	18.59	48.37	119.40	135.80	188.80	15.61	18.95	4.96	1.08
M14	16.90	4388.00	131.20	131.50	18.78	44.25	118.40	125.10	181.80	15.40	15.54	4.82	1.08
M15	17.62	4479.00	136.20	124.00	18.99	43.66	118.80	127.70	184.50	15.97	15.85	4.85	1.09
M16	17.51	4538.00	137.80	179.20	19.33	51.58	139.60	143.70	185.40	15.82	15.56	4.85	1.08
M17	16.86	4510.00	130.90	114.30	18.45	46.80	93.86	124.60	186.80	15.89	16.23	4.84	1.12
M18	17.25	4588.00	135.80	121.00	18.73	36.07	113.60	166.00	186.10	15.98	15.66	4.83	1.10
M19	16.69	4510.00	129.40	111.80	18.31	41.01	110.90	131.50	184.30	15.95	15.82	4.85	1.09
M20	16.80	4464.00	130.80	113.50	17.97	42.09	116.80	142.20	186.60	15.54	15.21	4.90	1.07
M21	17.18	4563.00	133.80	116.00	18.54	40.77	131.10	144.50	188.40	15.97	15.74	4.92	1.10
M22	16.89	4467.00	132.10	118.00	17.99	44.97	117.60	175.10	181.40	15.49	15.16	4.78	1.07
M23	17.24	4542.00	134.00	117.50	18.71	44.92	116.90	167.70	185.80	15.90	16.10	4.89	1.09
M24	16.85	4508.00	130.10	132.80	18.36	44.69	122.60	155.90	182.30	15.20	15.50	5.00	1.11
M25	15.81	4463.00	123.50	112.00	17.63	38.13	137.30	152.60	184.40	15.58	15.66	4.82	1.09
M26	16.04	4525.00	124.20	113.50	18.23	43.96	197.50	165.10	191.80	15.77	15.36	5.04	1.08
M27	17.11	4610.00	134.00	128.10	20.17	46.84	133.70	210.40	201.60	16.13	16.54	5.28	1.10
M28	16.94	4540.00	134.00	119.90	18.33	39.38	120.80	179.40	193.60	15.68	15.42	5.07	1.07
M29	17.51	4676.00	134.60	121.80	19.23	42.41	121.60	186.30	198.20	16.17	15.35	5.13	1.09
M30	16.61	4486.00	130.80	117.50	18.24	42.68	130.90	184.80	185.50	15.78	15.33	4.89	1.07
M31	16.47	4417.00	126.50	112.00	17.52	38.80	138.30	156.60	185.40	15.57	14.94	4.90	1.06
M32	17.19	4506.00	137.30	182.40	20.88	64.16	207.90	162.90	184.00	15.91	16.27	4.84	1.06
M33	17.53	4549.00	133.60	119.20	19.17	42.03	125.30	150.10	191.80	15.76	16.04	5.03	1.08
M34	17.41	4584.00	132.20	112.90	18.56	40.80	138.60	131.40	187.40	15.72	15.14	4.96	1.06
correlation coefficient r of ∑REE in the Wufeng Formation	0.524	0.529	–0.467	0.458	0.438	–0.426	–0.277	–0.409	0.693	0.526	0.603	0.673	0.514
correlation coefficient r of ∑REE in the Longmaxi Formation	0.536^b^	0.713^b^	0.307	–0.014	0.312	–0.014	–0.159	–0.249	0.752^b^	0.727^b^	0.587^b^	0.707^b^	0.670^b^

a The unit of test results for trace elements in the samples in the table is μg/g.

b Numbers indicate values higher than the significant correlation coefficient at the 99% significance level.

**Figure 6:**
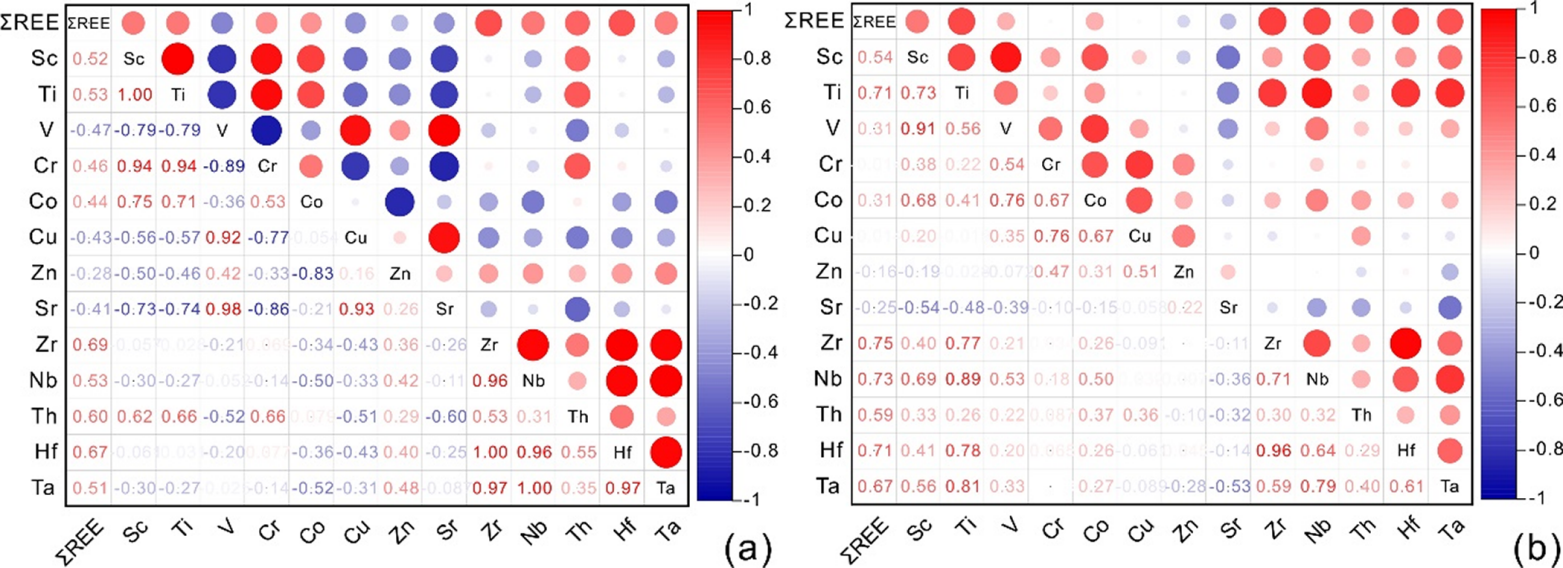
Heat map of Pearson's correlation analysis between ΣREE and trace elements in shale of Wufeng Formation (a) and Longmaxi Formation (b) in Well XX1.

As shown in Table 3 and Figure 6a, the ∑REE of the Wufeng Formation shale is fairly positively correlated with large ion lithophile elements such as Sc, Ti, Cr, Co, Zr, Nb, Th, Hf, and Ta and negatively correlated with the elements of V, Cu, Zn, and Sr. As shown in Table 3 and Figure 6b, the ∑REE of the Longmaxi Formation shale is significantly positively correlated with large ion lithophile elements such as Sc, Ti, Co, Zr, Nb, Th, Hf, and Ta and negatively correlated with the elements of Cr, Cu, Zn, and Sr. Based on the above analyses, it is believed that the sources of REEs in the Wufeng–Longmaxi Formation shales are similar, mainly terrigenous detritus, and some may come from the ocean.

### 5.4 Deposition Rate

The degree of differentiation of REEs can indirectly reflect the deposition rate. The principle is that suspended solids and detrital minerals, as the main carriers of rare earth elements, enter seawater, and the duration of their retention in seawater determines the degree of differentiation of rare earth elements.^42,43^ In general, the valence of ions of rare earth elements is positive trivalent, and the chemical properties of the elements are similar, maintaining a consistent pattern of element migration. However, some elements can undergo differentiation with changes in the seawater environment due to differences in their valence and adsorption properties, such as the differentiation of Ce and Eu with other elements and the differentiation of LREE and HREE.^42,43^ If the deposition rate is slow and the retention time in seawater is long, it is beneficial for REEs in suspended solids to have enough time to decompose, be adsorbed by clay minerals, and undergo a series of chemical reactions with organic matter, leading to strong differentiation of REEs and obvious loss and enrichment of light and heavy rare earth elements.^59,60^ Therefore, the degree of differentiation of REEs can be used to determine the deposition rate.^59,60^

The REEs were normalized by chondrite, and the slope of its partition curve could represent the degree of differentiation of the REEs. The higher the slope, the slower the deposition rate.^59,60^ The La_N_/Yb_N_ value can also be used to characterize the degree of differentiation of REEs.^44,45^ As shown in Table 2, the La_N_/Yb_N_ values of the Wufeng Formation samples in the study area range from 9.86 to 12.16, with an average value of 10.98. The La_N_/Yb_N_ values of the Longmaxi Formation samples range from 9.97 to 11.70, with an average of 10.59. The relatively close La_N_/Yb_N_ values reflect that the overall deposition rates of the Wufeng–Longmaxi Formations are not significantly different. Combined with the obvious rightward inclination of the chondrite-normalized REE partition curve, this indicates that the deposition rates of the Wufeng–Longmaxi Formations were generally low during the sedimentation period, reflecting the characteristics of the sedimentary area being far from the source area.

### 5.5 Properties of the Sediment Source

**Figure 7:**
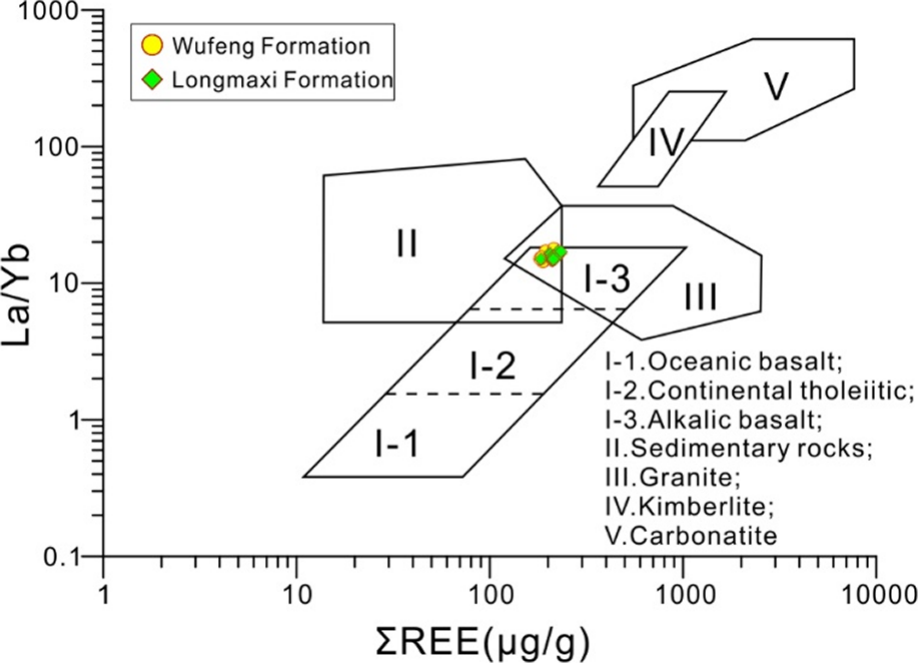
La/Yb−ΣREE diagram of the Wufeng–Longmaxi Formation shale in Well XX1.

REEs can retain the geochemical information from the sediment source area well, which is of great significance for tracking the sediment source.^61^ Generally, the chondrite-normalized REE partition curve of the upper crust is characterized by enrichment of light rare earth elements, deficit of heavy rare earth elements, and a negative Eu anomaly.^47^ The chondrite-normalized REE partition curve was carried out in samples from Well XX1 in the study area. As can be seen from the partition pattern diagram (Figure 3a,b), the Wufeng–Longmaxi Formations show a partition pattern of REEs that is basically consistent with that of the upper crust. The overall pattern is right-leaning, showing significant enrichment of light rare earth elements, a stable content of heavy rare earth elements, and an obvious negative Eu anomaly, indicating that its main provenance is from the upper crust. The distribution patterns of REEs in all samples are basically consistent, indicating good provenance stability. Previous studies used La/Yb–∑REE diagrams to determine the sources of sediments and the characteristics of the source area.^62^ It can be seen from Figure 7 that the samples of the Wufeng–Longmaxi Formations are relatively concentrated, located in the overlapping area of the sedimentary rock area, the granite area, and the alkalic basalt area, reflecting the characteristics of the source of the mixture and the diverse nature of the parent rock. Furthermore, it is suggested that the value of δEu can be used to determine the material source of the parent rock. It is generally believed that if the parent rock is granite, the sedimentary rocks mostly have negative Eu anomalies. If the parent rock is basalt, the sedimentary rocks have no negative Eu anomalies.^62^ The δEu in the study area is generally less than 1, with an average of 0.80, which is a typical negative anomaly. Therefore, it is inferred that the primary parent rock in the study area is granite. The study area is located in the northern Sichuan Basin of the Upper Yangtze Platform, which was covered by extensive epeiric sea at the end of the Ordovician.^36^ At the end of the Ordovician, during the strongest period of the Caledonian movement, the Upper Yangtze Platform was in a compressive tectonic setting, forming numerous uplifts around the Upper Yangtze Platform.^63^ These uplifts are exposed from the surface and are subjected to weathering and erosion, providing sedimentary materials for surrounding areas. Well XX1 is located among the “Chuanzhong Uplift” in the south, the “Xixiang Rising” archipelago in the northwest, and the “Hannan Old Land” in the north.^38,63^ Among them, the “Chuanzhong Uplift” and “Xixiang Uplift” at the end of the Ordovician are mainly due to the partial denudation of Cambrian and Ordovician strata in the Early Paleozoic, while the “Hannan Old Land” is not very clear, but it is certain that the Sinian Dengying Formation still exists today. Therefore, it can be inferred that the sediment parent rocks in the Well XX1 area are mainly early sedimentary rocks, and these early sedimentary rocks have characteristics of granite provenance.

### 5.6 Tectonic Setting Judgment

Previous studies believe that the geochemical characteristics of REEs under different tectonic backgrounds can be used to infer the tectonic environment at that time, and it is widely used to distinguish the tectonic setting of sedimentary rocks.^45,64^ Bhatia summarized the REE characteristics and related parameter ranges of greywacke under different tectonic background conditions, as shown in Table 4.^64^ However, previous studies have found that mud shale samples had a higher content of rare earth elements than greywacke samples,^65^ which cannot be directly compared and distinguished from the REE parameters of different tectonic backgrounds summarized by Bhatia.^64^ Condie found that under the same tectonic background, the total content of rare earth elements of mud shale samples was approximately 20% higher than that of greywacke samples.^65^ Therefore, the REE content of graywacke which deposited in the same period with the shale samples in Well XX1 can be obtained by dividing the REE value of shale samples in Well XX1 by the REE value of 1.2, namely, the corrected content. The corrected REE characteristic values can be compared with the REE characteristic parameters summarized by Bhatia to distinguish the tectonic environment of sedimentary basins.^64^

**Table 4:** Comparison of REE Characteristics between the Samples in the Study Area and the Greywackes in Sedimentary Basins with Different Tectonic Settings^a^

tectonic settings	provenance type	REE parameters						
		La	Ce	∑REE	La/Yb	L/H	La_N_/Yb_N_	δEu
oceanic island arc^b^	undissected magmatic arc	8 ± 1.7	19 ± 3.7	58 ± 10	4.2 ± 1.3	3.8 ± 0.9	2.8 ± 0.9	1.04 ± 0.11
continental island arc^b^	dissected magmatic arc	27 ± 4.5	59 ± 8.8	146 ± 20	11 ± 3.6	7.7 ± 1.7	7.5 ± 2.5	0.79 ± 0.13
Andean-type continental margin^b^	uplifted basement	37	78	186	12.6	9.1	8.5	0.6
passive margins^b^	craton interior tectonic highlands	39	85	210	15.9	8.5	10.8	0.56
XX1 well data (average)		47.97	91.09	212.59	15.77	9.58	10.66	0.8
XX1 well data (average) correction value		39.98	75.91	177.16	13.14	7.98	8.88	0.67
Wufeng Formation		45.61	84.97	199.64	16.25	9.65	10.98	0.79
correction value of Wufeng Formation		38.01	70.81	166.37	13.54	8.04	9.15	0.66
Longmaxi Formation		48.48	92.41	215.36	15.67	9.57	10.59	0.8
correction value of Longmaxi Formation		40.4	77.01	179.47	13.06	7.98	8.83	0.67

a The data were cited from the literature (Bhatia),^64^ with correction value = mean/1.2. Mean values in μg/g.

b Mean element ratios calculated from individual ratios.

From Table 4, it can be found that the ∑REE of shale in the Well XX1 area has an average value of 212.59 μg/g, with a corrected average value 177.16 μg/g, slightly lower than the average value of the active continental margin (186 μg/g). The average corrected values for La and Ce are 39.98 and 75.91 μg/g, respectively, which are close to the active continental margin and the passive continental margin. The average values of La/Yb, L/H, and La_N_/Yb_N_ are 15.77, 9.58, and 10.66, respectively, which are significantly different from the active continental margin and very close to the passive continental margin. The average value of δEu is 0.8, which is very close to the continental island arc (0.79 ± 0.13). The above analyses show that the tectonic setting of the study area is dominated by passive continental margin, and the provenance is mainly from the intracraton tectonic highlands, and there may be a cutting magmatic arc provenance from the continental island arc environment. The tectonic location of the study area is located near the northwest margin of the Yangtze Platform, among the “Chuanzhong Uplift”, “Xixiang Rising” archipelago, and “Hannan Old Land”,^63^ which may be affected by provenances from different tectonic backgrounds, resulting in complex REE geochemical characteristic parameters. This is consistent with the characteristics shown by the properties of the sediment source.

## 6 Conclusions

(1) The provenance supply or sedimentary-tectonic background may change during the Wufeng Formation shale deposition period; the Longmaxi Formation shows a relatively stable provenance supply and sedimentary-tectonic background. The distribution curve of rare earth elements in the shale of the study area shows a right-leaning pattern with an obvious negative Eu anomaly, which is characterized by typical crustal source sedimentation.

(2) The shale of the Wufeng Formation in the study area was mainly in the suboxic-anoxic conditions during the sedimentation period. During the sedimentation of shale in the Longmaxi Formation, the degree of water reduction is weakened, mainly under suboxic conditions. From the perspective of the TOC content, redox conditions may be an important factor in controlling organic matter enrichment.

(3) The overall deposition rate of the Wufeng–Longmaxi Formations in the study area is relatively low, which reflects that the depositional area is far from the provenance area. The source rocks of the shale samples are early sedimentary rocks with granite provenance characteristics. It is inferred that the tectonic setting of the study area is mainly the passive continental margin, and the provenance is mainly from the tectonic highlands in the craton. At the same time, there may be the source supply of cutting magma arcs from the continental island arc environment.

## Abbreviations

REEs: rare earth elements
∑REE: total content of REE
NASC: North American shale composite
LREE: light rare earth elements
HREE: heavy rare earth elements
TOC: total organic carbon
La_N_: chondrite-normalized values of lanthanum
Ce_N_: chondrite-normalized values of cerium
Pr_N_: chondrite-normalized values of praseodymium
Sm_N_: chondrite-normalized values of samarium
Eu_N_: chondrite-normalized values of europium
Gd_N_: chondrite-normalized values of gadolinium
Yb_N_: chondrite-normalized values of ytterbium
δEu: calculated value by Eu_N_/(Sm_N_ × Gd_N_)^0.5^
δCe: calculated value by Ce_N_/(La_N_ × Pr_N_)^0.5^
La_n_: North American shale composite normalized values of lanthanum
Ce_n_: North American shale composite normalized values of cerium
Nd_n_: North American shale composite normalized values of neodymium
Yb_n_: North American shale composite normalized values of ytterbium
Ce_anom_: cerium anomaly index calculated by lg[3(Ce_n_/(2La_n_+Nd_n_))]
|r|: correlation coefficient
